# Protein import into isolated pea root leucoplasts

**DOI:** 10.3389/fpls.2015.00690

**Published:** 2015-09-04

**Authors:** Chiung-Chih Chu, Hsou-min Li

**Affiliations:** Institute of Molecular Biology, Academia SinicaTaipei, Taiwan

**Keywords:** leucoplasts, plastid, root, protein import, translocon

## Abstract

Leucoplasts are important organelles for the synthesis and storage of starch, lipids and proteins. However, molecular mechanism of protein import into leucoplasts and how it differs from that of import into chloroplasts remain unknown. We used pea seedlings for both chloroplast and leucoplast isolations to compare within the same species. We further optimized the isolation and import conditions to improve import efficiency and to permit a quantitative comparison between the two plastid types. The authenticity of the import was verified using a mitochondrial precursor protein. Our results show that, when normalized to Toc75, most translocon proteins are less abundant in leucoplasts than in chloroplasts. A precursor shown to prefer the receptor Toc132 indeed had relatively more similar import efficiencies between chloroplasts and leucoplasts compared to precursors that prefer Toc159. Furthermore we found two precursors that exhibited very high import efficiency into leucoplasts. Their transit peptides may be candidates for delivering transgenic proteins into leucoplasts and for analyzing motifs important for leucoplast import.

## Introduction

Plastids are essential plant organelles responsible for functions ranging from photosynthesis and biosynthesis of amino acids and fatty acids to assimilation of nitrogen and sulfur (Leister, 2003; Sakamoto et al., 2008). All plastids are derived from embryonic proplastids and differentiate into different functional types in different tissues, for example chloroplasts in green tissues for photosynthesis, chromoplasts in petals and fruits for carotenoid pigment accumulation, and leucoplasts in non-green tissues for nutrient storage (Wise, 2006). To perform these specific functions, different plastid types require different proteins (Kleffmann et al., 2007; Brautigam and Weber, 2009; Barsan et al., 2012).

Although plastids have their own genome, most plastid proteins are encoded by the nuclear genome and synthesized in the cytosol as a larger precursor with an N-terminal extension called the transit peptide. Almost all of our current understanding of the molecular mechanism of plastid protein import is derived from studies with chloroplasts. Import across the chloroplast envelope membranes is mediated by the TOC and TIC (translocon at the outer/inner envelope membrane of chloroplasts) machinery. More than fifteen TOC and TIC components have been identified. Among them, Toc159 and Toc34 are receptors mediating the initial binding of precursor proteins to chloroplasts. Toc75 functions as the channel for protein translocation across the outer membrane. Major components of the TIC machinery includes Tic110 and the 1-MDa channel complex containing Tic20, Tic56, Tic100, and Tic214. Tic110 functions as the stromal site receptor for transit peptides and to act as a scaffold for tethering other translocon components located in the stroma. Hsp93, Hsp90C, and cpHsc70 are three chaperone motors important for the translocation of proteins into the stroma. Tic40 is a co-chaperone coordinating the actions of Tic110 and Hsp93 (for reviews see Li and Chiu, 2010; Shi and Theg, 2013; Paila et al., 2015).

The *Arabidopsis* Toc159 proteins are encoded by a four-gene family: Toc159, Toc132, Toc120, and Toc90. Toc159 and Toc132 are the major isoforms, with Toc90 and Toc120 as their functional redundant homologs, respectively. Toc159 preferentially binds transit peptides of photosynthetic proteins while Toc132 seems to prefer transit peptides of housekeeping proteins. Therefore, even though their substrate preference is not absolute, it is generally thought that Toc159 is the major receptor for photosynthetic proteins while Toc132 is important in mediating the import of non-photosynthetic housekeeping proteins into chloroplasts (Bauer et al., 2002; Ivanova et al., 2004; Kubis et al., 2004; Smith et al., 2004; Inoue et al., 2010; Bischof et al., 2011).

Leucoplasts are colorless, non-pigment-containing plastids. They are usually found in storage tissues and include the starch-storing amyloplasts, oil- and lipid-storing elaioplasts and protein-storing proteinoplasts (Wise, 2006). Most grain- and root-type food crops use leucoplasts to synthesize and store the nutrients that are used to feed the majority of the world population. However, despite the economic importance of leucoplasts, little is known about how proteins are imported into leucoplasts. It has been speculated that leucoplasts may use a similar basic import mechanism to that of chloroplasts (Jarvis and Lopez-Juez, 2013) but leucoplasts clearly have a preference for importing certain non-photosynthetic proteins (Wan et al., 1996; Yan et al., 2006; Primavesi et al., 2008). For example, only the transit peptide of the non-photosynthetic ferredoxin III and FtsZ precursors, but not that of the small subunit of RuBP carboxylase precursor (prRBCS), directs the import of GFP into leucoplasts in endosperms of transgenic wheat (Primavesi et al., 2008). Using leucoplasts isolated from caster seeds and chloroplasts isolated from pea leaves and the ratio of precursor to mature proteins after import as an assessment of import efficiency, it has been shown that prRBCS was imported preferentially into chloroplasts, while precursors to RuBP carboxylase activase (prRCA) and ferredoxin-NADP^+^-oxidoreductase (prFNR) were imported equally well into both plastids (Wan et al., 1996). Using leucoplasts and chloroplasts isolated from pea roots and leaves, respectively, it has been shown that prRBCS could not be imported into leucoplasts at all while two other non-photosynthetic precursors could (Yan et al., 2006). The plastid selectivity is determined by the transit peptide of each precursor as swapping of transit peptides resulted in interchanged plastid preference (Wan et al., 1995, 1996; Yan et al., 2006). It has been suggested that the different substrate preferences of Toc159 and Toc132 may also account for the import preferences of the two plastids (Yan et al., 2006; Li and Teng, 2013). Indeed, in *Arabidopsis*, Toc159 is about 15-fold higher in leaves than in roots while Toc132 is only about fivefold higher in leaves (Ivanova et al., 2004). Furthermore, the *toc159* knockout mutant has severe import defects in mesophyll-cell chloroplasts (Bauer et al., 2000) but imports proteins normally into root plastids (Yu and Li, 2001). On the other hand, *toc132 toc120* double mutations cause structural abnormalities in root plastids (Kubis et al., 2004), and the *toc132* mutant is a root gravitropism mutant (Stanga et al., 2009), supporting that Toc159 is more important for leaf chloroplasts and Toc132 is more important for root leucoplasts.

As a first step in characterizing the molecular mechanism of protein import into leucoplasts, we optimized the *in vitro* protein import system of isolated leucoplasts to improve import efficiency and to permit a more quantitative comparison between leucoplasts and chloroplasts. We found that leucoplasts have a different stoichiometry of translocon components compared to chloroplasts. Precursors we tested fell into at least three different degrees of preference toward chloroplasts in our *in vitro* import system.

## Materials and Methods

### Plastid Isolation and Plastid Number Counting

Leucoplasts and chloroplasts were prepared from pea seedlings (*Pisum sativum* cv. Green Arrow, De Bruyn Seed Co., Zeeland, MI, USA) grown at 20°C on vermiculite for 4–5 days in the dark, and for 7 days under a 12-h photoperiod with a light intensity of approximately 150 μmol m^-2^ s^-1^, respectively. Leucoplasts were prepared from pea roots as described (Bowsher et al., 1989) with modifications described below. Approximately 20∼30 g of roots were collected and washed twice with 100 mL homogenization buffer (50 mM Tricine-KOH, pH 7.9, 330 mM sorbitol, 1 mM MgCl_2_, 2 mM EDTA) in the presence of 1% BSA and reducing agents (2 mM ascorbic acid, 0.1 mM DTT, and 1.2 mM glutathione). The roots were cut into small pieces in another 100 mL of the same buffer, and were homogenized with a domestic blender by two 5-s pulses. The homogenate was filtered through four layers of Miracloth and centrifuged at 4,000 *g* for 3 min at 4°C. The leucoplast pellet was resuspended with 10 mL of homogenization buffer, underlaid with 10 mL 10% (v/v) Percoll^TM^ in TS buffer (50 mM Tricine-KOH, pH 7.9, 330 mM sorbitol), and centrifuged with a swing-bucket rotor at 4,000 *g* for 5 min at 4°C. The pellet was first washed with 30 mL TS buffer and again with 30 mL of import buffer (50 mM HEPES-KOH, pH 8.0, 330 mM sorbitol), by centrifugation at 4,000 *g* at 4°C for 3 min. The final pellet was gently resuspended with 300 μL of import buffer.

Chloroplast isolation from pea leaves was conducted as described (Perry et al., 1991) except 2 mM ascorbic acid, 0.1 mM DTT, and 1.2 mM glutathione were added to the grinding buffer used for homogenization. Isolated chloroplasts were adjusted to 1 mg chlorophyll mL^-1^ in import buffer.

To count the plastid number and estimate the plastid size, fractions of isolated leucoplast and chloroplast suspensions were counted in the Multisizer 3 Coulter Counter (Beckman Coulter) using a 30 μm-aperture tube. The same plastid preparations were subjected to BCA assays (Thermo) to determine the protein concentration. It is noted here that the sensitivity of the BCA assay is protein dependent (Smith et al., 1985) and since leucoplasts and chloroplasts have different protein compositions, which may affect the results of the determination. However, the assay should still be able to provide an estimation of the amount of proteins in the two plastids.

### Plasmid Construction and Translation of Precursors

The coding region of soybean mitochondrial alternative oxidase precursor (prAOX, accession number X68702) was amplified from a soybean leaf cDNA pool by the forward primer AOX-F1-*Xho*I (5′-gactcgagatgatgatgatgatgagccgc-3′) and the reverse primer AOX-R1-*Sal*I (5′-gcgtcgacttagtgataaccaattggagcagc-3′). The PCR products were digested with *Xho*I and *Sal*I and cloned into the *Xho*I/*Sal*I site of pSP72. The sequence of prAOX was confirmed by sequencing and the plasmid was named pSP72-prAOX. The expression of prAOX was under the control of the SP6 promoter. Plasmids encoding prRBCS, pea prTic40, prFd-protA_His_ (Smith et al., 2004), prPDH E1α (At1g01090), and prCpn10-2 (At3g60210) have been described (Teng et al., 2012). [^35^S]Met-labeled prPDH E1α was generated by *in vitro* transcription for synthesizing RNA followed by *in vitro* translation using the rabbit reticulocyte lysate system (Promega). All other precursors were synthesized using the TNT Coupled Wheat Germ Extract or Reticulocyte Lysate system (Promega).

### Protein Import and Post-Import Analyses

To compare import efficiency on an equal protein basis, 18 μL [^35^S]Met-labeled precursors were incubated with isolated plastids equivalent to 500 μg proteins in the presence of 3 mM ATP in import buffer in a final volume of 200 μL. To compare import efficiency on an equal plastid number basis, 113.67 μg leucoplast proteins and 500 μg chloroplast proteins were used instead. The import reactions were carried out at room temperature for 25 min and stopped by transferring to a new tube containing 1 mL cold import buffer. The plastids were pelleted at 3,000 g at 4°C for 3 min and resuspended in 200 μL import buffer. The leucoplast suspensions were underlaid with 1 mL 10% Percoll^TM^ (v/v) in import buffer and the chloroplast suspensions were laid on top of a 40% Percoll^TM^ (v/v) cushion and centrifuged in a swinging-bucket rotor at 2,900 *g* and 4°C for 6 min to isolate intact plastids. The plastids were washed once with import buffer. Thermolysin treatments of *in vitro* translation products and leucoplasts after import were performed as described (Perry et al., 1991), and intact leucoplasts were re-isolated as described above. Protein concentrations of the plastid samples were measured with the BCA kit (Thermo). Samples were analyzed by SDS-PAGE. Quantification of gel bands was performed using the Fuji FLA5000 PhosphorImager (Fujifilm).

### Isolation of cDNA Encoding Partial Pea Toc132AG and Generation of Antibodies against Pea Toc132AG and OEP24

Because the cDNA sequence of pea *Toc132* was not available in the NCBI database, the *Toc132* cDNA sequences from two closely related species, *Medicago truncatula* and *Glycine max*, were aligned and two highly conserved regions in the acidic domain (A domain) and GTPase domain (G domain) were chosen to design primers (Supplementary Figure S1A) for cloning. First-stand cDNA was synthesized from total pea-root RNA and used as templates to amplify the partial coding region of pea Toc132 A and G domains with the degenerate forward primer psToc132A-*Nde*I-F2 (5′-gggcatatggawsttggagatgacaagatagagg-3′) and the reverse primer psToc132G-*Xho*I-R1 (5′-gggctcgagtgcacttgcacgatcaaagctaaa-3′). The sequence without the primer regions (Supplementary Figure S1B) has been submitted to the GenBank and received the accession number KT033462. The PCR products were cloned into the pCR^TM^-Blunt II-TOPO^®^ (Invitrogen) to generate pTOPO-psToc132AG. After confirming the sequence, the DNA fragment of psToc132AG was excised by *Nde*I and *Xho*I from pTOPO-psToc132AG. Because the psToc132AG cDNA fragment has an endogenous *Xho*I site, only the longer psToc132 DNA fragment, in which *Xho*I cut at the *Xho*I site on the vector backbone, was subcloned into the *Nde*I and *Xho*I site of pET22b (Invitrogen) to generate the plasmid pET22b-psToc132AG-His_6_. The sequence was confirmed again and the pET22b-psToc132AG-His_6_ plasmid was transformed into the *Escherichia coli* strain BL21 (DE3) for protein induction. Protein expression of psToc132AG-His_6_ was induced by 1 mM IPTG at 37°C for 3 h, purified by TALON resins (CLONTECH Laboratories) with 50 mM imidazole, dialyzed with 50 mM Tris-HCl, pH 8.0, concentrated by Amicon Ultra-15 (Millipore) and used to raise the anti-Toc132 antibodies used in this study. During preparing the manuscript, another clone encoding a partial pea Toc132AG domains has been reported (Chang et al., 2014) and the amino acid sequence is different from the psToc132AG clone we obtained. Full-length pea OEP24 cDNA (EMBL accession number AJ001009) was amplified from a first-strand cDNA library of pea leaves and cloned into the *Nde*I/*Xho*I site of pET22b. OEP24 protein was then expressed and purified from *E. coli* and used for antibody production. Antibodies against other TOC and TIC proteins were prepared as described (Chou et al., 2003; Tu et al., 2004). Antibodies against cpHsc70 and Lhcb1 were purchased from Agrisera (AS08 348 and AS01 004, respectively), and the antibody against *Arabidopsis* plastid PGI was a gift of Dr. Jychian Chen.

## Results

### Optimization of the Protocol for Leucoplast Isolation

**FIGURE 1:**
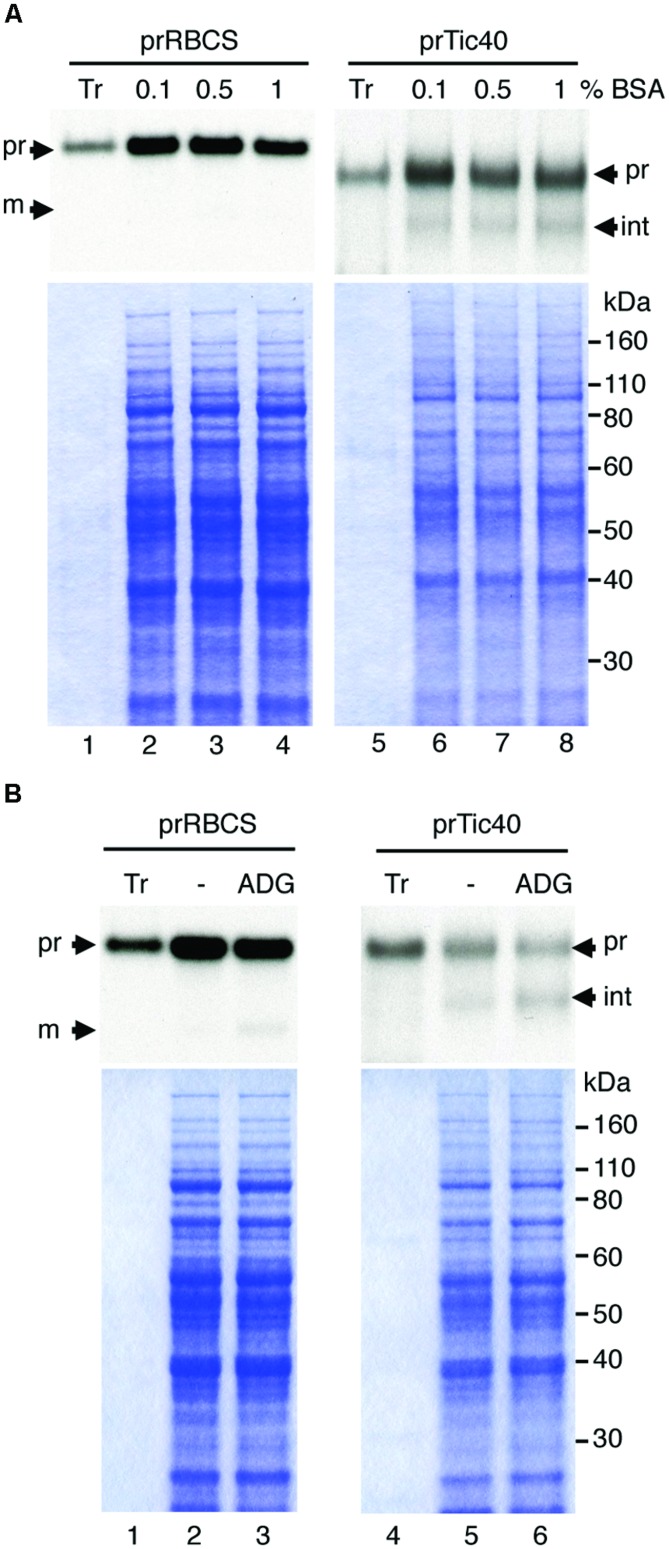
**Increasing BSA concentration and adding reducing agents to the homogenization buffer increases the import of prRBCS and prTic40 into leucoplasts. (A)** Roots of 4-days-old pea seedlings grown in the dark were harvested and homogenized in homogenization buffer (see Materials and Methods) containing 0.1, 0.5, or 1% BSA. Isolated leucoplasts (500 μg protein) were then incubated with *in vitro*-translated [^35^S]Met-prRBCS or [^35^S]Met-prTic40 under import conditions for 25 min. After import, intact leucoplasts were re-isolated and analyzed by SDS-PAGE. The gels were stained with Coomassie blue and dried for fluorography. Twenty micrograms of proteins were loaded. Tr, 1% equivalent of the *in vitro*-translated proteins used in each import reaction. **(B)** Same as **(A)** except the homogenization buffer was supplemented with 1% BSA, and with or without 2 mM ascorbic acid, 0.1 mM DTT, and 1.2 mM glutathione (ADG). pr, precursor form; int, imported intermediate form; m, imported mature form.

Several reports have described protein import into isolated leucoplasts (Wan et al., 1996; Yan et al., 2006). We first followed an established protocol to isolate leucoplasts from roots of pea seedlings grown for 4–5 days in the dark (Bowsher et al., 1989). Leucoplasts isolated from pea roots have been studied extensively. They are on average 2.5 μm in diameter and contain starch grains, plastoglobuli, and also a few lamellae-type structures (Emes and England, 1986; Bowsher et al., 1989). When we tested the import of several precursor proteins from our collection, we found only prTic40 was imported and only to a small degree (data not shown). We therefore sought to improve the import efficiency of isolated leucoplasts. Because EDTA was reported to have a protective effect on recovering intact chloroplasts (Somerville and Ogren, 1981) and BSA may protect plastids during isolation by limiting the activity of proteases (Seigneurin-Berny et al., 2008), we increased the concentrations of EDTA and BSA in the homogenization buffer. As shown in **Figure 1A**, the amount of processed Tic40 slightly increased and precursors associated with the leucoplasts slightly decreased with higher concentrations of BSA (**Figure 1A**, compare lanes 6–8). Precursors of Tic40 are processed twice upon import into chloroplasts, generating an intermediate and a mature form (Li and Schnell, 2006; Tripp et al., 2007). The leucoplast imported prTic40 we observed was mostly the intermediate form (**Figure 1A**, int). We also noticed that poor import occasionally occurred concomitant with dark brown leucoplast pellets. We surmised that some oxidative stress might have occurred during the isolation. It has been shown that pea roots contain a higher phenol oxidase activity than do pea leaves (Henry et al., 1979) and it is also possible that isolated leucoplasts are more sensitive to oxidative damages than do isolated chloroplasts. We therefore added reducing agents (2 mM ascorbic acid, 0.1 mM DTT, and 1.2 mM glutathione, designated as ADG in **Figure 1B**) to the homogenization buffer (Schulz et al., 2004; Aronsson and Jarvis, 2011). Indeed after adding the reducing agents, we no longer observed dark-brown leucoplast pellets and the import efficiency of prTic40 further increased (**Figure 1B**, lanes 5–6). Under the modified conditions, a very small amount of import could even be detected for prRBCS (**Figure 1B**, lane 3).

### Reducing the Number of Leucoplasts in Import Reactions Increases Import Efficiency

**FIGURE 2:**
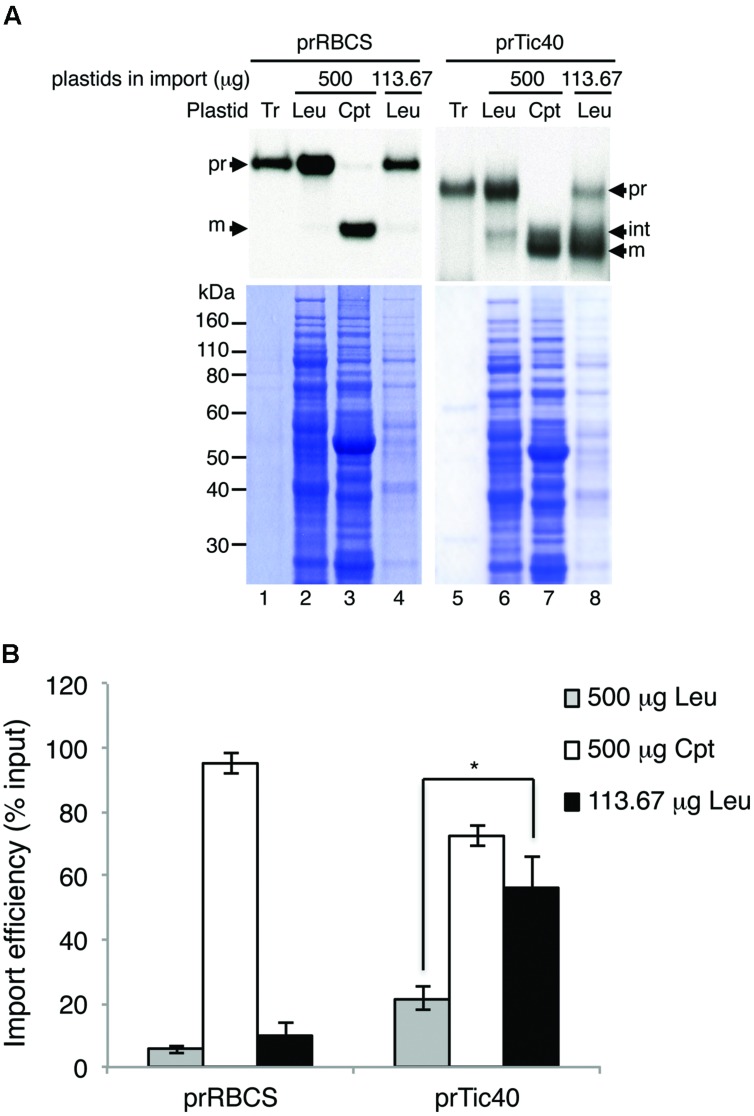
**Reducing the leucoplast concentration in import reactions increases import efficiency. (A)** Isolated leucoplasts (500 or 113.67 μg of proteins) and chloroplasts (500 μg of proteins) were incubated under import conditions with 18 μL *in vitro*-translated [^35^S]Met-prRBCS or [^35^S]Met-prTic40 and 3 mM ATP in import buffer in a final volume of 200 μL for 25 min. After import, intact leucoplasts and chloroplasts were re-isolated and analyzed by SDS-PAGE. The gels were stained with Coomassie blue and dried for fluorography. 4% of the plastids in each import reaction were loaded. Tr, 1% equivalent of the *in vitro*-translated proteins used in each import reaction. Cpt, chloroplasts; Leu, leucoplasts; pr, precursor form; int, imported intermediate form; m, imported mature form. **(B)** Imported proteins in experiments as shown in **(A)** were quantified and the import efficiencies were calculated. Import efficiency was defined as % of [^35^S]-labeled precursor proteins used that were found as imported mature or intermediate proteins in re-isolated plastids. The values have been corrected for the difference in number of methionine residues among the various forms. Data shown are mean ± SD of three independent experiments, ^∗^*p <* 0.05 (Student’s *t*-test). The *p*-value of 500 μg Cpt versus 113.67 μg Leu of prTic40 import is 0.095.

After optimizing the leucoplast isolation conditions, we next tried to compare the import behaviors of various precursors into leucoplasts and chloroplasts. An equal amount of total proteins (Yan et al., 2006) or an equal number of plastids (Wan et al., 1996) was used as the basis when comparing import efficiencies of different plastids. To determine the number of plastids, the isolated leucoplasts and chloroplasts were counted using the Multisizer 3 Coulter Counter (Beckman Coulter). We used chloroplasts isolated from 7-days-old seedlings for robust import and to minimize the age difference between the leucoplast and chloroplast samples. From three independent preparations of leucoplasts and chloroplasts, the average size of the isolated leucoplasts and chloroplasts was estimated to be 1.81 ± 0.21 μm and 3.24 ± 0.23 μm, respectively (**Table 1**). The same plastid preparations were then used for protein concentration determination and the average protein content per plastid was calculated to be 1.85 ± 0.63 and 8.13 ± 1.42 pg/plastid for leucoplasts and chloroplasts, respectively (**Table 1**). We then used protein content as an estimate of plastid numbers throughout our analyses. For example, 500 μg of plastid proteins would represent approximately 2.71 × 10^8^ leucoplasts and 6.15 × 10^7^ chloroplasts. For import into chloroplasts, we used 500 μg of chloroplasts (∼6.15 × 10^7^ plastids) in a 200 μL import reaction. We then either used 500 μg of leucoplasts for comparison on an equal protein basis or 113.67 μg of leucoplasts (∼6.15 × 10^7^ plastids) for comparison on an equal plastid number basis. After import, 4% of each import reaction was analyzed by SDS-PAGE. Interestingly, a lower concentration of leucoplasts (113.67 μg/reaction) in the import reactions resulted in a significant increase of prTic40 import efficiency (**Figure 2A**, compare lanes 6–8), compared to when 500 μg/reaction were used, which was the amount we used for **Figure 1**. Using this lower leucoplast input, the import efficiency of prTic40 was almost equal to its efficiency into chloroplasts and most of the imported proteins were in the mature form (**Figure 2A**, lanes 7 and 8). The import efficiency of prRBCS into leucoplasts was also slightly increased when a lower amount of leucoplasts was used, but the efficiency was still much lower than its efficiency into chloroplasts (**Figures 2A**, lanes 2–4, **2A**). From these data, we decided to compare the import of different precursors into leucoplasts and chloroplasts on an equal plastid number basis in all subsequent analyses.

**Table 1:** The sizes and protein contents of the isolated plastids.

Plastid type	Plastid size (μm)	Protein content (pg/plastid)
Chloroplasts (7-days-old light-grown leaves)	3.24 ± 0.23	8.13 ± 1.42
Leucoplasts (4-days-old dark-grown roots)	1.81 ± 0.21	1.85 ± 0.63

### Mitochondrial Precursor prAOX is not Imported into Isolated Leucoplasts

**FIGURE 3:**
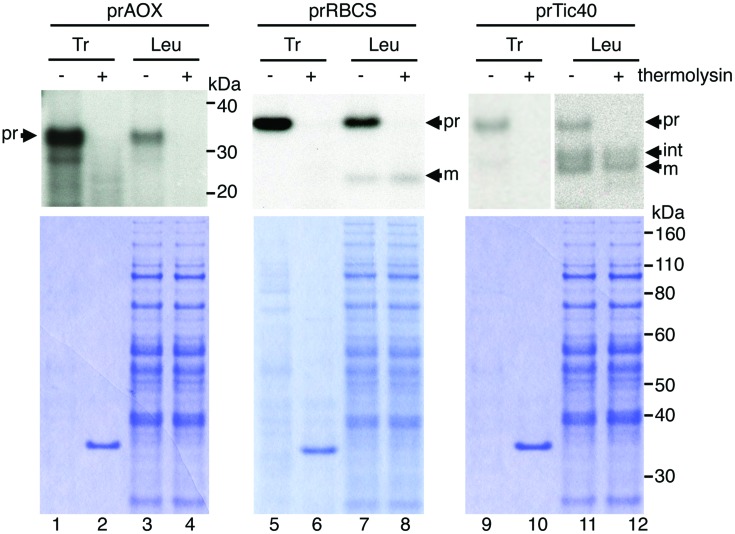
**The mitochondrial precursor protein prAOX was not imported into isolated leucoplasts.** Isolated leucoplasts (113.67 μg) were incubated with *in vitro*-translated [^35^S]Met-prAOX, [^35^S]Met-prRBCS, or [^35^S]Met-prTic40 under import conditions for 25 min. After import, the leucoplasts were treated with 0.2 mg/mL thermolysin for 30 min on ice, and intact leucoplasts were re-isolated and analyzed by SDS-PAGE. 4% of the plastids in each import reaction were loaded. The gels were stained with Coomassie blue and dried for fluorography. Tr, 1% equivalent of the *in vitro*-translated proteins used in each import reaction. Leu, leucoplasts; pr, precursor form; int, imported intermediate form; m, imported mature form.

The leucoplast preparations were reported to have about 1% contamination of mitochondria (Emes and England, 1986; Bowsher et al., 1989). Furthermore, it has been reported that chloroplast precursors can be imported into isolated pea mitochondria (Rudhe et al., 2002). To verify that the import we observed was not due to mis-sorting of plastid precursors to the mitochondria in the leucoplast preparation, we isolated cDNA clone encoding the soybean mitochondrial alternative oxidase precursor protein (prAOX), which is one of the most widely used precursor proteins for studying protein import into isolated plant mitochondria. The precursor form of prAOX is about 36 kDa. After being imported into isolated mitochondria, it is processed to a 32-kDa mature protein, which is protected exogenously added protease (Whelan et al., 1995; Rudhe et al., 2002; Lister et al., 2007). [^35^S]Met-labeled prAOX was synthesized and used in the leucoplast import assays as prTic40 and prRBCS. If the import we observed was due to import of prRBCS and prTic40 into the mitochondria in the leucoplast preparation, then a mitochondrial precursor like prAOX should import equally well or even better. As shown in **Figure 3**, although some prAOX signals were detected after import, these signals were degraded after treating the leucoplasts with thermolysin. No imported mature protein was detected even after over-exposure of the fluorograph (**Figure 3**, lanes 3 and 4). In contrast, both prRBCS and prTic40 produced mature proteins after import, and these mature proteins were thermolysin resistant, indicating that they were inside the leucoplasts (**Figure 3**, lanes 7, 8, 11, and 12).

### Most TOC/TIC Components are more Abundant in Chloroplasts than in Leucoplasts

**FIGURE 4:**
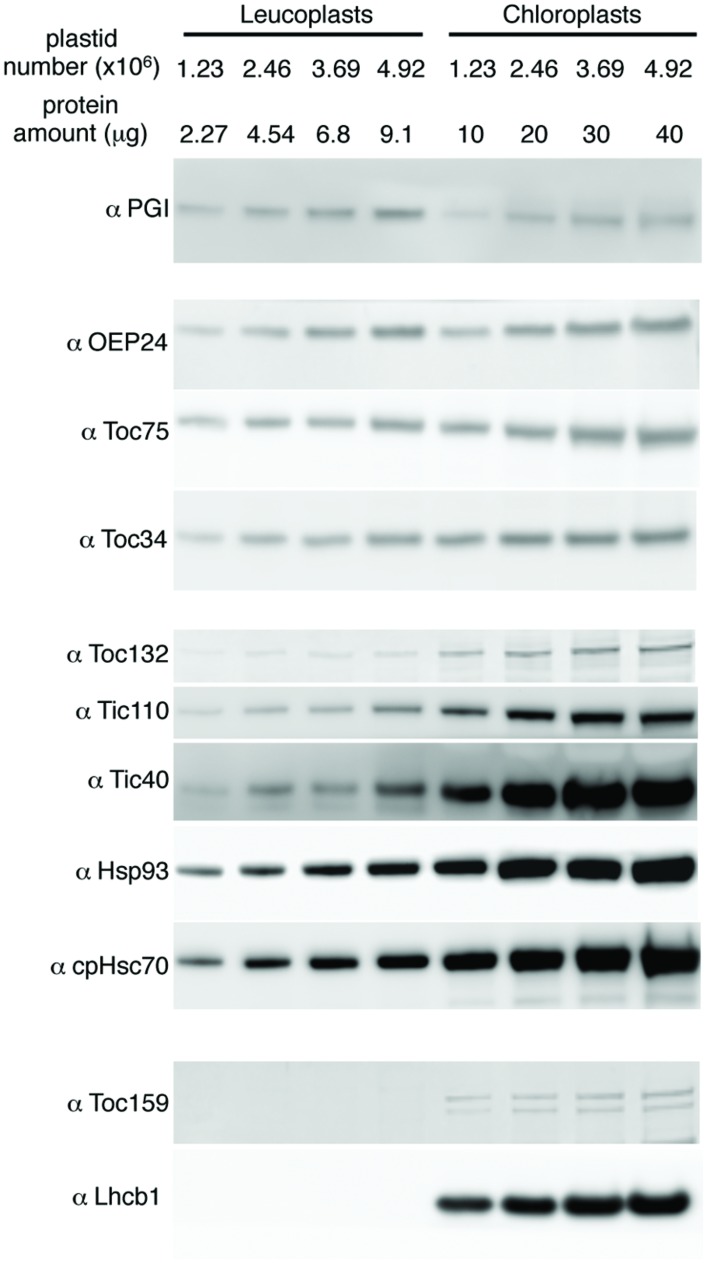
**Translocon protein abundance in leucoplasts and chloroplasts compared on an equal number of plastids basis.** Various amounts of isolated leucoplasts and chloroplasts were analyzed by SDS-PAGE and immunoblotting with antibodies indicated at left. The amount of protein loaded in each lane and the equivalent number of plastids are indicated above the lanes.

To investigate the translocon composition of leucoplasts, representative translocon proteins were analyzed by immunoblots. We used phosphoglucoisomerase (PGI), a plastid enzyme in the starch biosynthesis pathway and expressed in both leaves and roots, as a non-translocon stromal reference protein. We also used OEP24, a non-translocon plastid outer membrane protein expressed in both leaves and roots (Pohlmeyer et al., 1998), for a general assessment of the amount of the envelope proteins. When compared on the basis of an equal number of plastids, the amount of PGI in chloroplasts was similar or slightly lower than that in leucoplasts (**Figure 4**). The amount of OEP24 and the translocon proteins Toc75 and Toc34 was about 1.5x to 2.5x higher in chloroplasts than in leucoplasts. In comparison, abundances of Toc132, Tic110, Hsp93, cpHsc70, and Tic40 were much higher in chloroplasts. Toc159 is known to be extremely sensitive to protease and is easily degraded during plastid isolation (Bolter et al., 1998). In chloroplasts, we detected, in addition to the full-length Toc159, at least one lower molecular weight band that is most likely a partially degraded Toc159 fragments. We could not detect any full-length Toc159 either in our leucoplast preparation (**Figure 4**) or when roots were directly ground up in SDS-PAGE sample buffer in liquid nitrogen (data not shown). Very low amounts of smaller fragments were detected in leucoplasts (data not shown) but our antibody was not specific enough to exclude cross-reactivity with other Toc159 members. Nonetheless, even if Toc159 is present in the leucoplasts of pea roots, it is much less abundant than in chloroplasts. In addition, no chlorophyll *a/b* binding protein of photosystem II (Lhcb1) could be detected, suggesting that our leucoplasts were not contaminated with chloroplasts.

### Import of prFd-protA_His_, prPDH E1α, and prCpn10-2 into Leucoplasts

**FIGURE 5:**
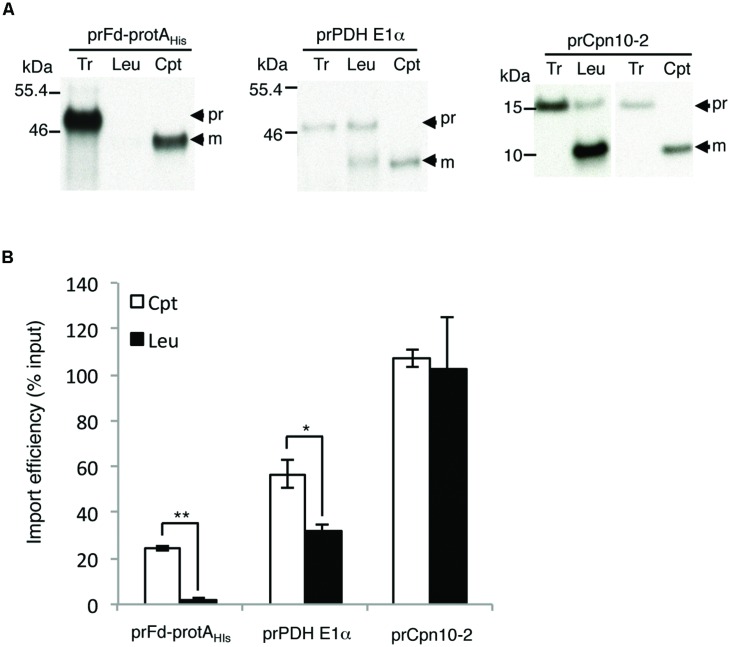
**Precursor proteins show different plastid preferences. (A)** Isolated leucoplasts (113.67 μg protein) and chloroplasts (500 μg protein) were incubated with various *in vitro*-translated [^35^S]Met-precursors under import conditions for 25 min. After import, intact leucoplasts and chloroplasts were re-isolated and analyzed by SDS-PAGE. The gels were stained with Coomassie blue and dried for fluorography. 4% of the plastids in each import reaction were loaded. Tr, 1% (for prFd-protA_His_ and prPDH E1α) or 1.2% (for prCpn10-2) equivalent of the *in vitro*-translated proteins used in each import reaction. Cpt, chloroplasts; Leu, leucoplasts; pr, precursor form; m, imported mature form. **(B)** Imported mature proteins in experiments as shown in **(A)** were quantified and the import efficiencies were calculated. Import efficiency was defined as % of [^35^S]-labeled precursor proteins used that were found as mature proteins in re-isolated plastids. The values have been corrected for the difference in number of methionine residues between the precursor and mature forms. Data shown are mean ± SD of three independent experiments, ^∗^*p <* 0.05, ^∗∗^*p <* 0.001 and the *p*-value of prCpn10-2 Cpt versus Leu is 0.67 (Student’s *t-*test).

We had determined that pea root leucoplasts had a lower amount of Toc132 and almost no Toc159. If the interactions with these Toc159 family members contribute to plastid preference, a precursor that prefers Toc159 should import much better into chloroplasts, while a precursor that prefers Toc132 should exhibit a smaller difference. Our data with prRBCS indeed support this (**Figure 2B**). We further tested another two precursors that have been directly demonstrated to select between Toc159 and Toc132: the transit peptide of ferredoxin precursor (prFd) prefers Toc159 while the transit peptide of pyruvate dehydrogenase E1α subunit precursor (prPDH E1α) prefers Toc132 (Ivanova et al., 2004; Smith et al., 2004; Inoue et al., 2010). For prFd, we used the construct prFd-protA_His_, which contains ferredoxin transit peptide fused to Staphylococcal protein A (protA; Smith et al., 2004). As shown in **Figure 5**, prFd-protA_His_ imported well into chloroplasts but was nearly undetectable in leucoplasts, similar to the results for prRBCS (**Figure 2**). In comparison, although prPDH E1α also imported better into chloroplasts, its import was at least half as strong in leucoplasts in our *in vitro* system.

We further tested several additional precursors but did not find any precursors with higher import efficiency into leucoplasts than into chloroplasts in our system. However, we did find several precursors similar to prTic40 that exhibited no import bias between leucoplasts and chloroplasts in our *in vitro* system. One example was the precursor of chaperonin 10-2 (prCpn10-2), which imported very well into both chloroplasts and leucoplasts (**Figure 5B**). The import pathways prCpn10-2 and prTic40 used may be partially different from those of prRBCS and prPDH E1α.

## Discussion

We performed initial quantifications of translocon components of pea root leucoplasts. Considering that the diameter of chloroplasts we used is about 1.7 times that of leucoplasts, the chloroplast surface area should be about three times that of leucoplasts. However, the amount of chloroplast Toc75 and Toc34 is only about twice that in leucoplasts. Therefore, the density of Toc75 and Toc34 is not necessarily higher in chloroplasts. If the non-translocon protein OEP24 is present in about the same density in chloroplasts and leucoplasts, then the density of Toc75 and Toc34 in the two plastids would also be similar. On the other hand, other essential translocon components, like Tic110, Hsp93 and cpHsc70, are clearly less abundant in leucoplasts. We did not analyze the amount of other essential components like Tic20 and Tic56 (Kikuchi et al., 2013; Köhler et al., 2015) due to unavailability of antibodies recognizing the pea orthologous well and their amounts in leucoplasts remain to be determined. However, based on our current data, it is likely that similar numbers of Toc75 and Toc34 subunits are coupled with lower numbers of other translocon components in leucoplasts. This composition may reflect that, under normal physiological conditions, the protein import demand of leucoplasts is not as high as young chloroplasts. Once precursors are bound to the TOC complex, leucoplasts can afford a lower import rate than chloroplasts.

It has been proposed that the precursor selectivity between leucoplasts and chloroplasts may also be due to the distinct substrate preference of Toc159 versus Toc132 (Yan et al., 2006; Li and Teng, 2013). We therefore tested the three precursors that have been directly shown to prefer one receptor to the other. In agreement with the much higher abundance of Toc159 in leaf chloroplasts, prRBCS and prFd-protA_His_, which show a preference for Toc159, also imported much better into chloroplasts. Toc132 is still more abundant in chloroplasts but is present in leucoplasts at a reasonable amount. And indeed, prPDH E1α, which shows a preference for Toc132, had a leucoplast import efficiency about 50% that of chloroplasts in our current *in vitro* system. Unfortunately the transit peptide of prPDH E1α is the only transit peptide that has been shown biochemically to prefer Toc132 over Toc159. The importance of Toc132 in leucoplast import will need to be tested with more precursors with clear preferences for Toc132.

We did not find any precursors with higher import efficiency into leucoplasts than into chloroplasts in our system. It is possible that our leucoplast *in vitro* import system is still not as optimized as that of chloroplasts. However, compared to prRBCS/prFd and prPDH E1α, we found a third group of precursors that have similar import efficiencies into both plastids in our current *in vitro* system. Previous reports have indicated that prFNR, prRCA and the precursor of leucoplast pyruvate kinase also imported equally well into the two plastids (Wan et al., 1995, 1996). Although in those reports leucoplasts and chloroplasts were isolated from different species, it is likely that these precursors share some import characteristics with prTic40 and prCpn10-2 that we analyzed here. Unlike precursors that clearly prefer Toc159 or Toc132, the import of these precursors did not decline in leucoplasts. They may use some novel component present in similar amounts in the two plastids or they may simply bypass the need for the Toc159 family protein and use Toc34 and Toc75 directly. However, it is also possible that these precursors import into leucoplast through distinct pathways. It would be interesting to further study their import mechanism and identify the motifs in their transit peptides that confer efficient leucoplast import.

The transit peptide of prRBCS is the most widely used transit peptide for directing passenger proteins into plastids in transgenic plants, even when the passenger proteins were meant for non-green plastids like leucoplasts. For example, the prRBCS transit peptide was used to deliver the bacterial carotene desaturase into rice grain leucoplasts for the development of the Golden Rice (Ye et al., 2000; Paine et al., 2005). As shown here and other reports (Wan et al., 1996; Yan et al., 2006; Primavesi et al., 2008), the import efficiency of this transit peptide into leucoplasts is rather poor. The precursors that exhibited equally high efficiency into both chloroplasts and leucoplasts in our *in vitro* system, like prTic40 and prCpn10-2, offer promising potential that their transit peptides can confer better import of passenger proteins in transgenic plant leucoplasts than the prRBCS transit peptide. They could be valuable tools in manipulations of crops in which leucoplasts provide the major nutrient source.

## Author Contributions

C-CC and H-mL designed the experiments and prepared the manuscript. C-CC performed the experiments.

## Conflict of Interest Statement

The authors declare that the research was conducted in the absence of any commercial or financial relationships that could be construed as a potential conflict of interest.
