# Supplementary material for: Protein import into isolated pea root leucoplasts
Source: Front Plant Sci. 2015 Sep 4;6:690. doi: 10.3389/fpls.2015.00690 (PMC4560022; doi:10.3389/fpls.2015.00690)
Supplement: Supplementary file 1 [file Presentation_1.PDF]

Supplementary Material

Protein import into isolated pea root leucoplasts

Chiung-Chih Chu and Hsou-min Li*

* **Correspondence:** Dr. Hsou-min Li: mbhmli@gate.sinica.edu.tw

Supplemental figures S1A

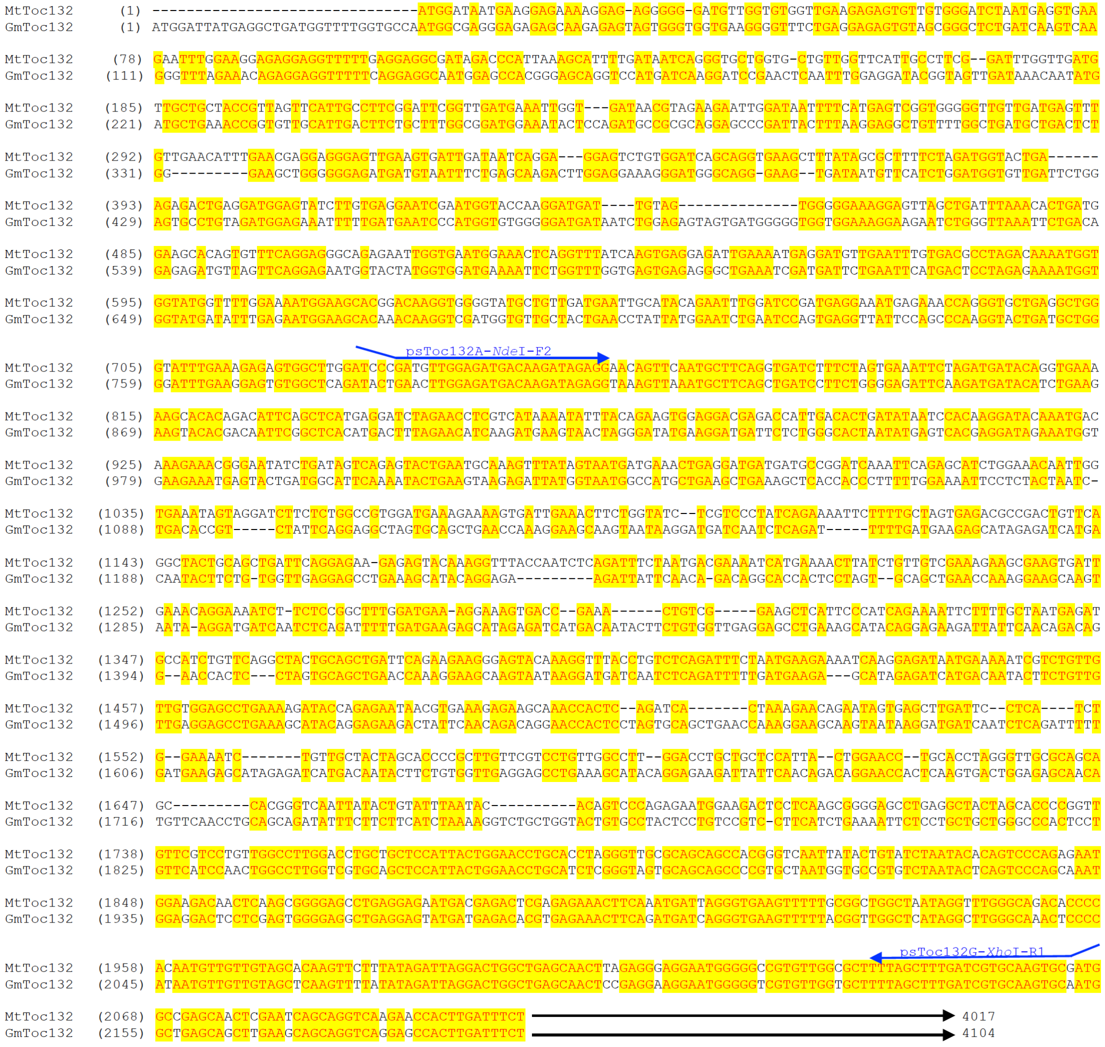

Supplemental figures S1B

```
atToc159     (1)  MDSKSVTPEPTNPFYASSGQSGKTYASVVAAAAAAAADKEDGGAVSSAKELDSSSEAVSGNSDKVGADDL
atToc132     (1)  ----------------------------------------------------------------------
psToc132AG   (1)  ----------------------------------------------------------------------

atToc159    (71)  SDSEKEKPNLVGDGKVSDEVDGSLKEDSTTPEATPKPEVVSGETIGVDDVSSLSPKPEAVSDGVGVVEEN
atToc132     (1)  -----------------------------------------------------------MGDGTEFVVRS
psToc132AG   (1)  ----------------------------------------------------------------------

atToc159   (141)  KKVKEDVEDIKDDGESKIENGSVDVDVKQASTDGESESKVKDVEEEDVGTKKDDEGESELGGKVDVDDKS
atToc132    (12)  DREDKKLAEDRISDEQVVKN----ELVR---SDEVRDDNEDEVFEEAIGSENDEQEEE------------
psToc132AG   (1)  ----------------------------------------------------------------------

atToc159   (211)  DNVIEEEGVELTDKGDVIVNSSPVESVHVDVAKPGVVVVGDAEGSEELKINADAETLEVANKFDQIGDDD
atToc132    (63)  -EDPKR---ELFESDDLPLVETLKSSMVEHEVEDFEEAVGDLDETS------------------------
psToc132AG   (1)  ----------------------------------------------------------------------

atToc159   (281)  SGEFEPVSDKAIEEVEEKFTSESDSIADSSKLESVDTSAVEPEVVAAESGSEPKDVEKANGLEKGMTYAE
atToc132   (105)  -------------------SNEGGVKDFTAVGESHGAGEAEFDVLATKMNGDKGEGGGGGSYDKVESSLD
psToc132AG   (1)  ----------------------------------------------------------------------

atToc159   (351)  VIKAASAVADNGTKEEESVLGGIVDDAEEGVKLNNKGDFVVDSSAIEAVNVDVAKPGVVVVGDVEVSEVL
atToc132   (156)  VVDTTENATSTNTN------------GSN---LAAEHVGIENGKTHSFLGNGIASP----KNKEVVAEVI
psToc132AG   (1)  ----------------------------------------------------------------------

atToc159   (421)  ETDGNIPDVHNKFDPIGQGEGGEVELESDKATEEGGGKLVSEGDSMVDSSVVDSVDADINVAEPGVVVVG
atToc132   (207)  PKDDGIEEPWNDGIEVDNWEERVDGIQTEQEVEEGEGTTENQFEKRTEEEVVEGEGTSKNLFEKQTEQDV
psToc132AG   (1)  --------------------------------------------------QCNASGDPYGEIQNDSAHGD

atToc159   (491)  AAKEAVIKEDDKDDEVDKTISNIEEPDDLTAAYDGNFELAVKEISEAAKVEPDEPKVGVEVEELPVSESL
atToc132   (277)  VEGEGTSKDLFENGSVCMDS----E---------------------------SEAERNGETGAAYTSNIV
psToc132AG  (21)  LEPNGEIFIEMNDETIDTDIS-------------------------------HGDTDGKEMGISDTQKTE

atToc159   (561)  KVGSVDAEEDSIPAAESQFEVRKVVEGDSAEEDENKLPVEDIVSSREFSFGGKEVDQEPSGEGVTRVDGS
atToc132   (316)  TNASGDNEVSSAVTSSPLEESSSGEKGETEGDSTCLKPEQHLASSPHSYPESTEVHSNSGSPGVTSREHK
psToc132AG  (60)  RKDYGNDEIKDDDVGSSAEHPEIGDTG--GFFSVVD--ERNVIETAGSSSLSEIFFATEMPAVQTTVTNS

atToc159   (631)  ESEEETEEMIFGSSEAAKQFLAELEKASSGIEAHSDEANISNNMSDRIDGQIVTDSDEDVDTEDEGEEKM
atToc132   (386)  PVQSANGGHDVQSP--------------Q----PNKELEKQQSSRVHVDPEITENSHVETEPEVVSSVSP
psToc132AG (126)  EEESMNVDRSKVSN-------------------EESQGNTENLSVVGEPIKILENNTIEKQMNQITQVQN
                                                                    A domain <-|-> G domain
atToc159   (701)  FDTAALAALLKAATGGGSSEGGNFTITSQDGTKLFSMDRPAGLSSSLRPLKPAAAPRANRSNIFSNSNVT
atToc132   (438)  TESRSNPAALPPARPAG-----------------------LGRASPLLEPASRAPQQSRVNGNGSHNQFQ
psToc132AG (177)  SEFVSSSGKSVANSTTLVHPAG------------------LGSAAPLLKPAPRVVQQVRANYTVSNTPSQ

atToc159   (771)  MADETEINLSEEEKQKLEKLQSLRVKFLRLLQRLGHSAEDSIAAQVLYRLALLAGRQAG-----QLFSLD
atToc132   (485)  QAEDSTTTEADEHDETREKLQLIRVKFLRLAHRLGQTPHNVVVAQVLYRLGLAEQLRGRNGSRVGAFSFD
psToc132AG (229)  KVEDSSTMEAEEYDETREKLQMIRVRFLRLANRFGQTPHNVVVAQVLYRLGLAEQLRGRNGGRVGA----

atToc159   (836)  AAKKKAVESEAEGNEELIFSLNILVLGKAGVGKSATINSI------→ 1503
atToc132   (555)  RASAMAEQLEAAGQDPLDFSCTIMVLGKSGVGKSATINSI------→ 1206
psToc132AG (295)  ----------------------------------------
```

Supplemental Figure S1. Sequence alignment of Toc132. (A) The cDNA sequences of Toc132 from *Medicago truncatula* (MtToc132, MTR_2g030630) and *Glycine max* (GmToc132, LOC100776898) were aligned using the AlignX software (Vector NTI Advance 9.1.0 version, Life Technologies). The positions of the primers used for amplification of pea Toc132 were indicated by the blue arrows. **(B)**The acidic domain and partial GTPase domain of atToc159 (AT4G02510), atToc132 (AT2G16640), and the partial pea Toc132 A and G domains we isolated (GeneBank accession no. KT033462) were aligned using the AlignX software. The A and G domains were defined as Bauer et al. (2000).

LITERATURE CITED

Bauer J, Chen K, Hiltbunner A, Wehrli E, Eugster M, Schnell D, Kessler F (2000) The major protein import receptor of plastids is essential for chloroplast biogenesis. Nature **403:** 203-207
